# Valid and reliable diagnostic performance of dual-energy CT in anterior cruciate ligament rupture

**DOI:** 10.1007/s00330-023-09720-y

**Published:** 2023-05-12

**Authors:** Di Liu, Ping Hu, Zi-Jun Cai, Wen-Hao Lu, Lin-Yuan Pan, Xu Liu, Xian-Jing Peng, Yu-Sheng Li, Wen-Feng Xiao

**Affiliations:** 1 Department of Orthopedics, Xiangya Hospital, Central South University, Changsha, 410008 Hunan China; 2 Department of Radiology, Xiangya Hospital, Central South University, Changsha, 410008 Hunan China; 3 National Clinical Research Center for Geriatric Disorders, Xiangya Hospital, Central South University, Changsha, 410008 Hunan China

**Keywords:** Radiography, dual-energy scanned projection, Tomography, x-ray computed, Anterior cruciate ligament, Rupture, Diagnosis

## Abstract

**Objectives:**

To determine whether dual-energy CT (DECT) can be used to accurately and reliably detect anterior cruciate ligament (ACL) rupture.

**Materials and methods:**

Participants with unilateral ACL rupture were prospectively enrolled, and the bilateral knees were scanned by DECT. A tissue-specific mapping algorithm was applied to improve the visualization of the ACLs. The 80-keV CT value, mixed-keV CT value, electron density (Rho), and effective atomic number (Z_eff_) were measured to quantitatively differentiate torn ACLs from normal ACLs. MRI and arthroscopy served as the reference standards.

**Results:**

Fifty-one participants (mean age, 27.0 ± 8.7 years; 31 men) were enrolled. Intact and torn ACLs were explicitly differentiated on color-coded DECT images. The 80-keV CT value, mixed-keV CT value, and Rho were significantly lower for the torn ACLs than for the intact ACLs (*p* < 0.001). The optimal cutoff values were an 80-keV CT value of 61.8 HU, a mixed-keV CT value of 60.9 HU, and a Rho of 51.8 HU, with AUCs of 98.0% (95% CI: 97.0–98.9%), 99.2% (95% CI: 98.6–99.7%), and 99.8% (95% CI: 99.6–100.0%), respectively. Overall, DECT had almost perfect reliability and validity in detecting ACL integrity (sensitivity = 97.1% [95% CI: 88.1–99.8%]; specificity = 98.0% [95% CI: 89.5–99.9%]; PPV = 98.0% [95% CI: 93.0–99.8%]; NPV = 97.1% [95% CI: 91.7–99.4%]; accuracy = 97.5% [95% CI: 94.3–99.2%]). There was no evidence of a difference between MRI and DECT in the diagnostic performance (*p* > 0.99).

**Conclusion:**

DECT has excellent diagnostic accuracy and reliability in qualitatively and quantitatively diagnosing ACL rupture.

**Clinical relevance statement:**

DECT could validly and reliably diagnose ACL rupture using both qualitative and quantitative methods, which may become a promising substitute for MRI to evaluate the integrity of injured ACLs and the maturity of postoperative ACL autografts.

**Key Points:**

*• On color-coded DECT images, an uncolored ACL was a reliable sign for qualitatively diagnosing ACL rupture.*

*• The 80-keV CT value, mixed-keV CT value, and Rho were significantly lower for the torn ACLs than for the intact ACLs, which contributed to the quantitative diagnosis of ACL rupture.*

*• DECT had an almost perfect diagnostic performance for ACL rupture, and diagnostic capability was comparable between MRI and DECT.*

## Introduction

The anterior cruciate ligament (ACL) is an important elastic structure that dynamically maintains normal knee kinematics in concert with other ligaments, muscles, and surrounding tissues. ACL rupture, however, is a common knee injury due to increased engagement in sports activities or strenuous work, and the incidence will continue to rise [1-4]. In clinical practice, ACL rupture is conventionally treated with various surgical reconstruction techniques to restore knee stability and functional integrity, particularly in active participants who wish to return to sports [5, 6]. In addition, ACL tears are associated with an increased risk of secondary injury to the knees, as well as the accelerated development of posttraumatic osteoarthritis (PTOA) [7-10]. Therefore, an accurate diagnosis plays a crucial role in the treatment and rehabilitation of participants with torn ACLs.

Although MRI is regarded as the gold standard for the noninvasive diagnosis of ACL rupture, it has disadvantages and limitations in the context of acute trauma, as well as in participants with specific contraindications (e.g., magnetic implants, pacemakers, claustrophobia, and obesity) [11-13]. Dual-energy CT (DECT), therefore, has become a promising substitute for MRI in the evaluation of ACL integrity. As an advanced imaging technology, DECT has the ability to differentiate soft tissues (e.g., ligaments and tendons) from other articular structures based on the differential attenuation that occurs at various energy levels [14, 15]. These material- and energy-specific images are preferable and valuable for clinical applications. With the advances that have been made in the development of postprocessing algorithms, specific materials can be identified and color-coded on monochromatic images for easy visual detection of ligamentous injuries [14, 15]. For conventional images, however, the image quality of DECT is lower than that of MRI; therefore, it is necessary to compare measurable values to improve the diagnostic reliability and accuracy of DECT for the evaluation of ACL injuries [14-16]. DECT also has inherent advantages over conventional CT, including improved imaging of osseous injuries, reduced artifacts from limb motion, abbreviated acquisition time, and reduced cost.

The primary purpose of the present study was to evaluate the qualitative and quantitative value of DECT for the diagnosis of ACL rupture, operating on the hypothesis that DECT is of comparable quality and reliability to MRI for the clinical diagnosis of ACL rupture.

## Materials and methods

### Study design and participants

This prospective study was a diagnostic trial, and the protocol for the present study was approved by the Institutional Review Board. Written informed consent was obtained from all participants. Between January 2022 and October 2022, consecutive participants were prospectively enrolled in the present study. Eligibility criteria included age over 14 years, nonpregnant status, unilateral ACL injury history, and positive physical examinations. Participants with meniscal or chondral lesions concomitant with ACL rupture were eligible as well. The exclusion criteria were as follows: bilateral ACL injury, no indication for ACL reconstruction, multiligament injury, contraindications to CT or MRI, pregnancy, rerupture after primary ACL reconstruction, revision ACL reconstruction, or other pathological changes in the knee joints.

### DECT imaging and reconstruction protocol

**Fig. 1:**
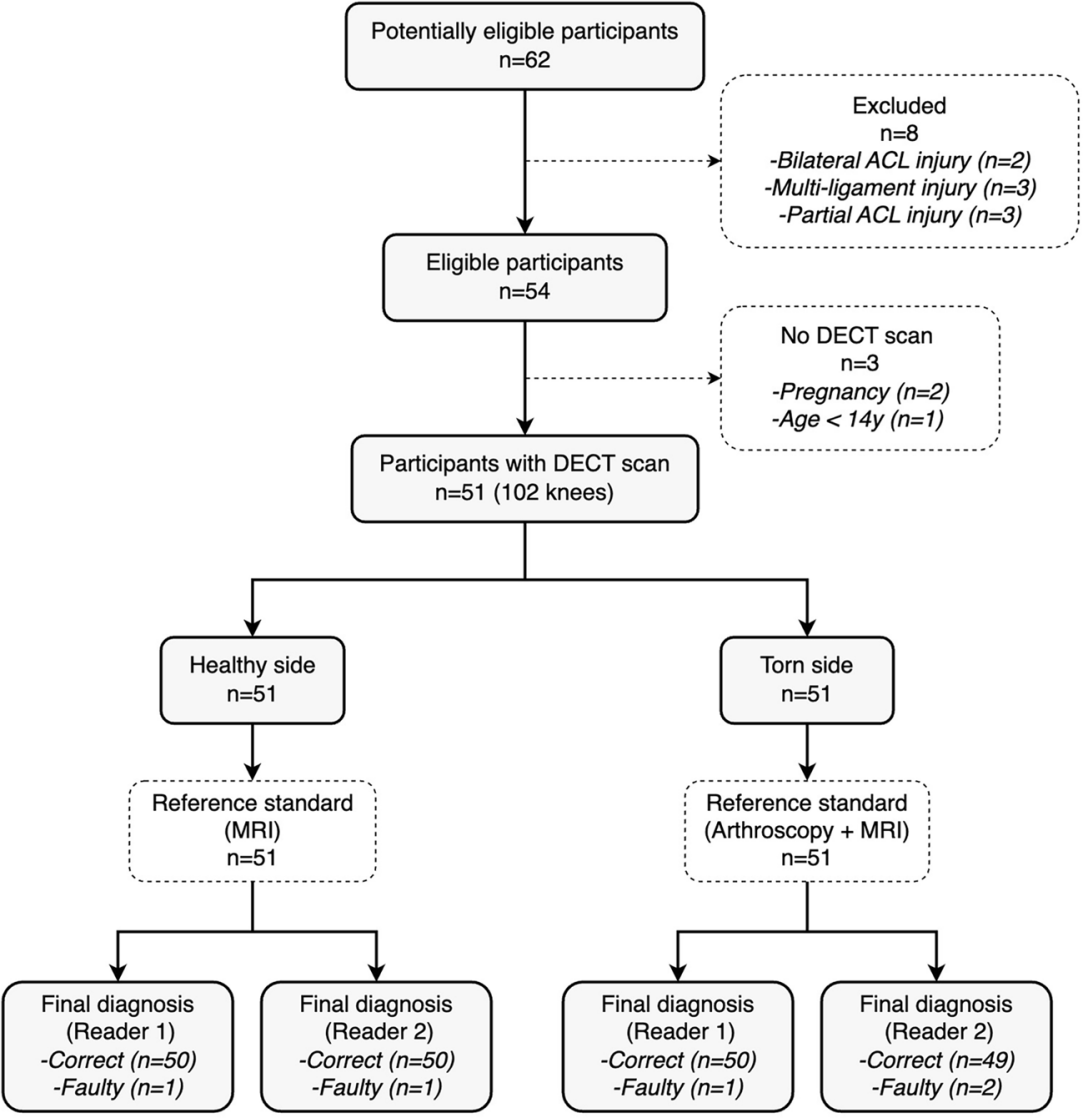
Study flow diagram

Both MRI and DECT examinations were performed on the bilateral knees of eligible participants (Fig. 1). DECT scans were conducted using a dual-source CT scanner in dual-energy mode (Somatom Drive; Siemens Healthcare), and all participants were shielded from scatter radiation with lead protective clothing. The injured and contralateral knees were scanned simultaneously using two X-ray tubes with different kilovoltage settings (tube A: 80 kV, 210 mAs; tube B: Sn140 kV, 105 mAs). The same imaging protocol was used for all participants, as follows: 1.0 mm slice thickness; 0.5 s rotation time; 32 × 0.6 mm detector collimation; and 0.7 helical pitch. The CT dose index volume (CTDIvol) was 6.0 mGy with automatic tube current modulation, resulting in an approximate dose-length product of 192 mGy · cm for a scan length of 32 cm.

Image reconstruction was performed on a Syngo workstation (VB20A; Siemens Healthcare) by a board-certified radiologist (P.H.) using a soft-tissue window (window width, 400; window level, 40). Axial, coronal, sagittal, and oblique sagittal images were reconstructed at a slice thickness of 0.75 mm, a slice increments of 0.5 mm, pixel size of 0.5 × 0.5 mm, matrix of 512 × 512, and field of view (FoV) of 250 mm, using the dual-energy kernel for soft tissues (Qr40). The bone marrow-specific algorithm facilitated the differentiation between torn and normal ACLs through continuous attempts. Thus, this algorithm was selected to color grayscale images, in which the normal ACLs were highlighted in black and dark red while the injured ACLs were almost uncolored. Oblique sagittal images from the normal dual-energy mode (50%:50% mixed keV, monochromatic and color-coded), monoenergetic plus (mono +) mode (80 keV, monochromatic and color-coded), and Rho/Z mode (color-coded) were chosen for the visualization and measurement of the ACLs. In mono + mode, DECT images were obtained at 80 keV based on readers’ experience and preference for appropriate visualization with better contrast and less noise.

### DECT analysis

The images were evaluated and measured on a Syngo workstation (VB20A; Siemens Healthineers) independently and in random order by the orthopedic surgeon (W.F.X. [reader 1]), with 14 years of experience in orthopedics and sports medicine, and the radiologist (X.J.P. [reader 2]), with 11 years of experience in musculoskeletal radiology. All identifying participant information was removed from the DECT and MRI datasets, and readers were blinded to participants’ demographic and clinical data, including name, sex, age, side and cause of injury, and time from injury to DECT scan. The image evaluation was repeated after 2 weeks.

**Fig. 2:**
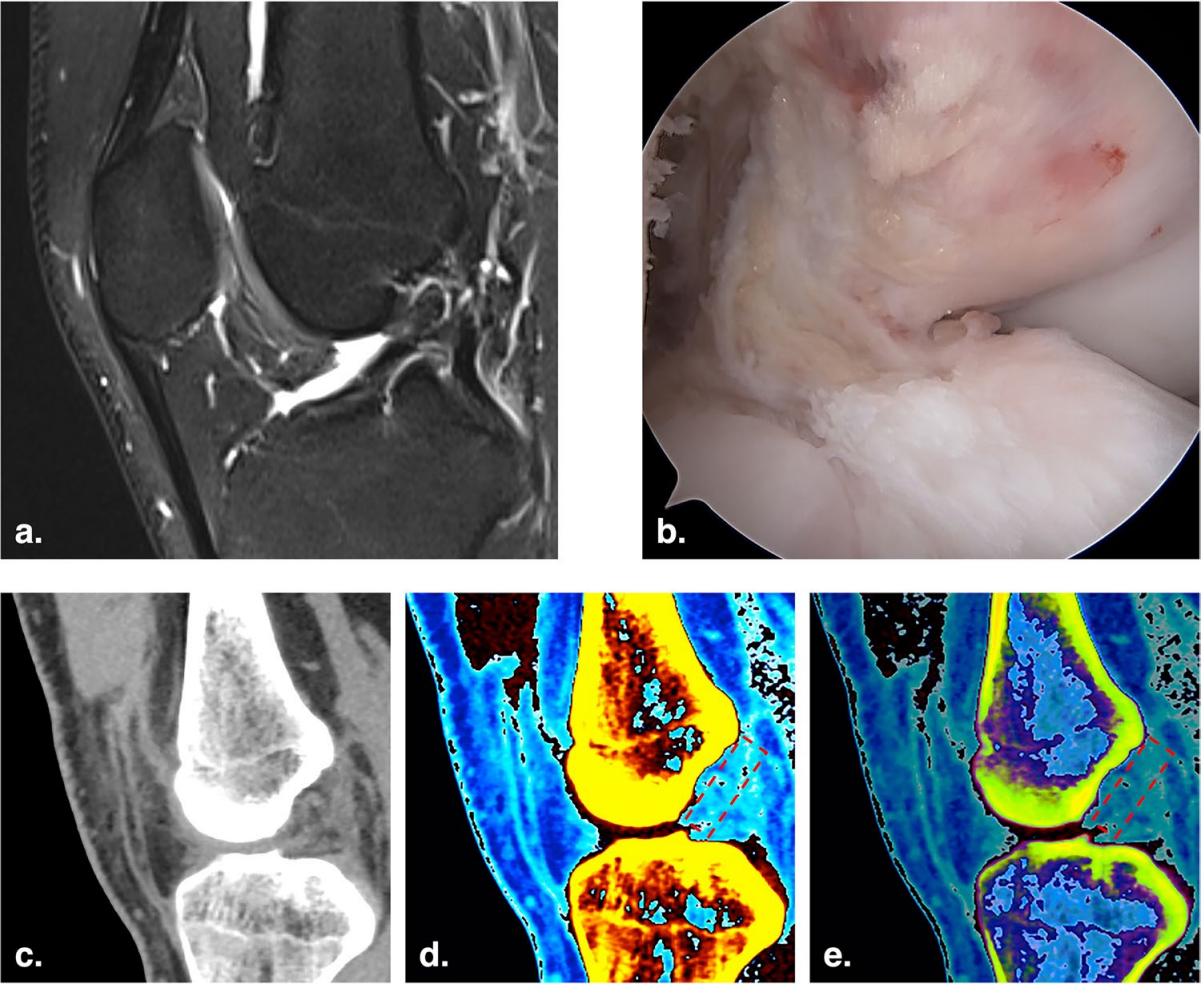
Images of a 22-year-old man with right ACL rupture. **a** Sagittal T2-weighted fat-saturated MRI image of the right knee. **b** Arthroscopic image of the torn right ACL. **c** Monochromatic DECT image with normal mode (mixed keV) on the oblique sagittal plane. **d** and** e** Color-coded DECT images with mono + mode (80 keV) and Rho/Z mode (mixed keV) on the oblique sagittal plane, respectively. The torn ACL was not colored in the anatomic position (red dotted rectangle)

During each ACL reconstruction, the injured knee was evaluated arthroscopically to confirm ACL rupture. The contralateral knees served as healthy controls; their health status was confirmed by no history of trauma, negative physical examinations, and intact ACLs on the MRI examinations (Fig. 2).

The quality of MRI and DECT images for the assessment of ACLs was scored on a 5-point Likert scale as follows: 1 = poor; 2 = slightly acceptable; 3 = fairly acceptable; 4 = good; and 5 = almost excellent.

**Fig. 3:**
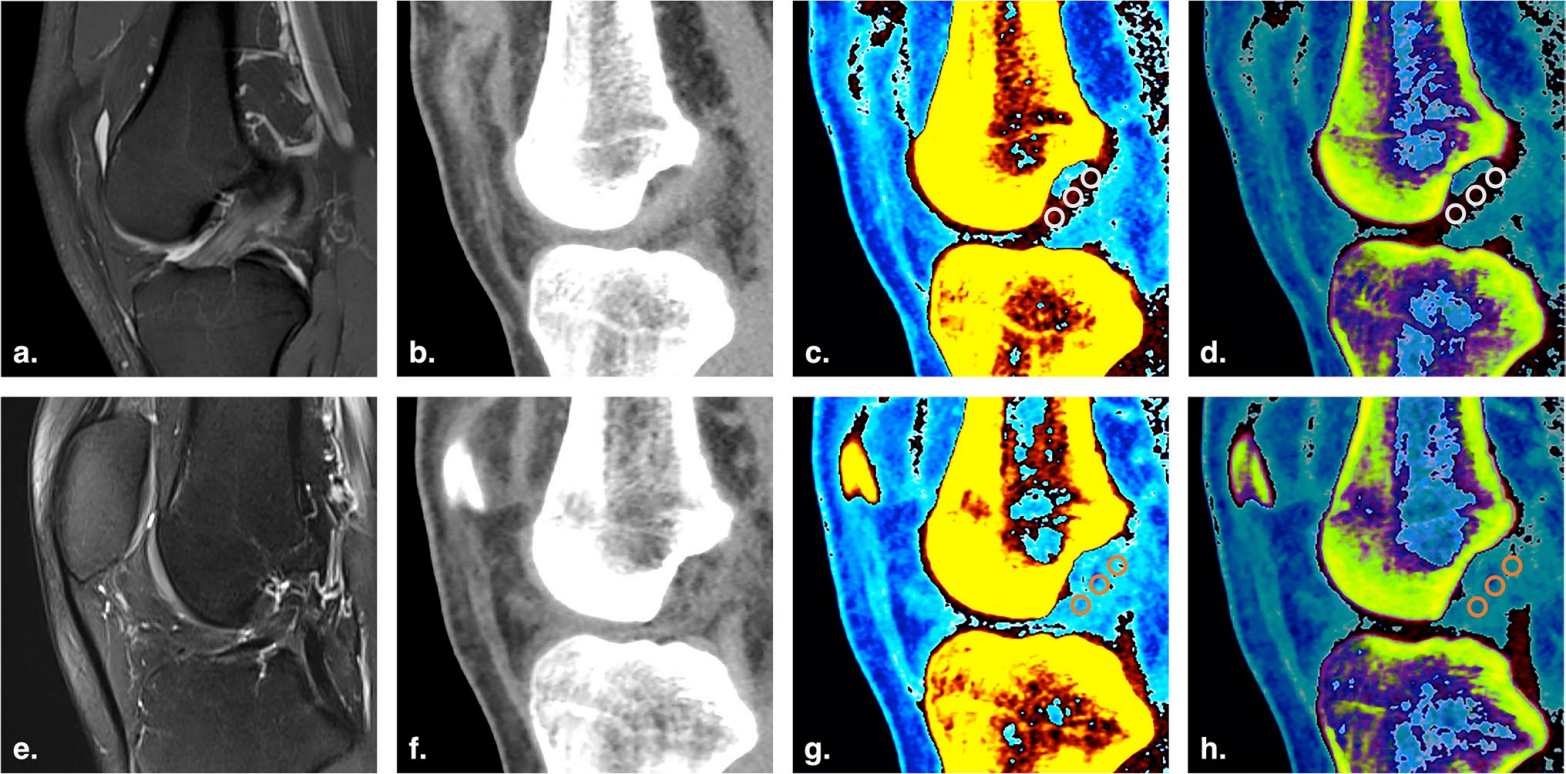
Images of a 25-year-old man with left ACL rupture. **a** and** e** Sagittal proton density-weighted and T2-weighted fat-saturated MRI images of the right and left knees, respectively. **b** and** f** Oblique sagittal plane, monochromatic DECT images with normal mode (mixed keV) of the right and left knees, respectively. **c** and** g** Oblique sagittal plane, color-coded DECT images with mono + mode (80 keV) of the right and left knees, respectively. **d** and** h** Oblique sagittal plane, color-coded DECT image with Rho/Z mode (mixed keV) of the right and left knees, respectively. The intact ACL was colored black and dark red in the anatomic position (**c** and** d**). The torn ACL was not colored (**g** and** h**). Three circular ROIs were set on the proximal, middle, and distal sites of the normal ACL (**c** and** d**) and torn ACL (**g** and** h**)**,** respectively

The mixed-keV CT value, 80-keV CT value, electron density (Rho), and effective atomic number (Z_eff_) were the measurable values used as the quantitative measurements for tissue characterization [17]. These measurements taken from both the torn and contralateral sides were compared in normal dual-energy, mono + , and Rho/Z modes. On each of the freely selected 3 oblique sagittal images, 3 circular regions of interest (ROIs), each 0.1 cm^2^, were selected at equidistant intervals (proximal, middle, and distal) on the intact and torn ACLs. Subsequently, the values were automatically displayed on the DECT images. The mixed-keV CT values were measured in normal dual-energy mode, the 80-keV CT values were measured using the mono + method, and the Rho and Z_eff_ values were measured using the Rho/Z method (Fig. 3).

Based on the visualized DECT images and measurable values, the two readers made their final diagnoses as follows: ANR, absolutely not ruptured; PNR, probably not ruptured; UCI, uncertain for ACL integrity; LR, likely ruptured; and DR, definitely ruptured. For intact ACLs, MRI images served as the gold standard, and the ANR option was defined as the correct diagnosis, whereas for injured ACLs, arthroscopic findings served as the gold standard, and the DR option was defined as the correct diagnosis. Finally, the diagnostic results were independently verified by an orthopedic investigator who was not involved in patient enrollment or surgery.

### Statistical analysis

The participants’ demographic characteristics were appropriately documented by descriptive statistics. The Mann‒Whitney *U* test and Student’s *t* test were used to verify the differences in the 80-keV CT value, mixed-keV CT value, Rho, and Z_eff_ between the torn and intact ACL groups. The diagnostic validity of DECT imaging was evaluated using the sensitivity, specificity, likelihood ratio (LR), positive predictive value (PPV), negative predictive value (NPV), and receiver operating characteristic (ROC) curve. Logistic regression analysis and ROC curve analysis were performed to evaluate the efficacy of DECT imaging and measurable values as predictors for ACL rupture. An area under the curve (AUC) was calculated, and Youden’s index was used to identify the optimal cutoff values for the 80-keV CT value, mixed-keV CT value, Rho, and Z_eff_ for the diagnosis of ACL ruptures. The AUC was interpreted as follows: fail to poor (0.50 ≤ AUC < 0.70); acceptable (0.7 ≤ AUC < 0.80); good (0.8 ≤ AUC < 0.90); and outstanding (0.9 ≤ AUC ≤ 1.0) [18]. Diagnostic reliability was evaluated based on consistency and intraclass correlation coefficient (ICC). The ICC was used to evaluate the intra- and interobserver agreement for the measurable values using the two-way random model and was interpreted as follows: poor agreement (ICC < 0.40); fair to good agreement (0.40 ≤ ICC ≤ 0.75); and excellent agreement (ICC > 0.75) [19, 20]. The diagnostic performance of DECT and MRI for ACL integrity was compared using McNemar’s test. Statistical analyses were conducted using SPSS software (version 26.0; IBM Corp.), with the threshold for statistical significance set at *p* < 0.05. For sample size calculation, the sensitivity and specificity were predefined at 75% and 90%, respectively, based on a previous study [21]. The prevalence of ACL ruptures in the target population was 50%; therefore, a sample size of 51 participants was required to obtain 90% power at a significance level of 0.05 to evaluate the diagnostic performance of DECT for ACL injuries. Sample size and post-hoc power analysis were performed using PASS software (version 15.0.5; NCSS).

## Results

### Participant characteristics

The main demographic features of the participants were summarized in Table 1. A total of 102 knees from 51 participants with ACL tears (31 male/20 female; 29 left/22 right knees; mean age, 27.0 ± 8.7 years [range, 15–47 years]; and mean body mass index [BMI], 24.9 ± 3.8 kg/m^2^ [range, 18.8–36.7 kg/m^2^]) were included. The median time from injury to DECT scan was 2.0 weeks (range, 1.0–8.6 weeks). The median time interval between MRI and DECT scans was < 1.0 days.

**Table 1:** Main participant demographics

Age, y^*^	27.0 ± 8.7 (15–47)
Sex, *n*	
Male	31
Female	20
BMI, kg/m^2*^	24.9 ± 3.8
Injured side, n	
Left	29
Right	22
Time from injury to DECT examination, weeks^#^	2.0 (1.0–8.6)
Time interval between MRI and DECT, days^#^	< 1.0
Concomitant injury, n (%)	
Meniscal tear	37 (72.5%)
Cartilage lesion	22 (43.1%)

*BMI*, body mass index; *n*, number

^*^Data expressed as mean ± standard deviation

^#^Data expressed as median (*p*_25_–*p*_75_)

### Quality of DECT and MRI images for viewing ACLs

The two readers independently graded the DECT and MRI images (Table 2) and found that although those from MRI had almost excellent quality, DECT images ranged from good to excellent quality for the visualization of ruptured and intact sides, with uncolored ACLs and black and dark red ACLs, respectively.

**Table 2:** DECT and MRI scores for the visualization of ACLs

	Ruptured ACLs	Contralateral ACLs
DECT		
Average of readers^*^	4.8	5.0
Reader 1^*^	4.8	4.9
Reader 2^*^	5.0	5.0
MRI		
Average of readers^*^	5.0	5.0
Reader 1^*^	5.0	5.0
Reader 2^*^	5.0	5.0

^*^Data expressed as mean value

### Quantitative analysis between torn and intact ACLs

**Fig. 4:**
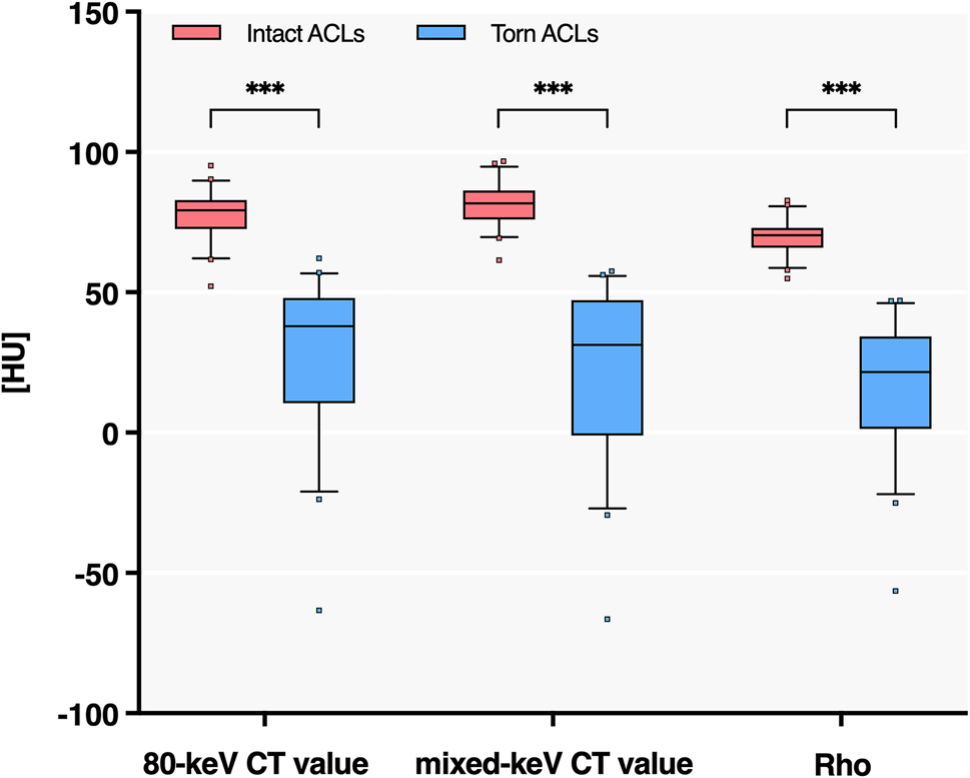
Comparisons of the 80-keV CT value, mixed-keV CT value, and Rho between intact and ruptured ACLs. ^***^ indicates *p* < 0.001

The 80-keV CT value, mixed-keV CT value, Rho of torn ACLs were found to be significantly lower than those of the healthy controls (*p* < 0.001) (Fig. 4, Table 3).

**Table 3:** Measurement of 80-keV CT value, mixed-keV CT value, Rho, and Z_eff_ for DECT

	Ruptured ACLs	Intact ACLs	*p* value
80-keV CT value, HU			
Overall^#, *^	37.9 (10.5–48.0)	77.9 ± 7.7	** < 0.001**
Reader 1^#, *^	41.4 (16.2–49.4)	77.5 ± 9.1	** < 0.001**
Reader 2^#, *^	35.1 (5.6–46.6)	78.3 ± 7.3	** < 0.001**
Optimal cut-off value	61.8		
Mixed-keV CT value, HU			
Overall^#, *^	31.3 (− 1.0–47.2)	81.4 ± 7.2	** < 0.001**
Reader 1^#, *^	34.5 (3.9–48.4)	80.9 ± 8.4	** < 0.001**
Reader 2^#, *^	28.0 (− 3.6–46.4)	81.9 ± 7.4	** < 0.001**
Optimal cut-off value	60.9		
Rho, HU			
Overall^#, *^	21.6 (1.4–34.3)	70.0 ± 6.1	** < 0.001**
Reader 1^#, *^	22.5 (2.1–36.6)	69.1 ± 7.1	** < 0.001**
Reader 2^#, *^	20.5 (–1.3–34.2)	70.9 ± 6.4	** < 0.001**
Optimal cut-off value	51.8		
Z_eff_, HU/Z			
Overall^#, *^	7.59 ± 0.21	7.71 (7.61–7.75)	**0.029**
Reader 1^#, *^	7.59 ± 0.23	7.72 (7.63–7.78)	**0.032**
Reader 2^*^	7.58 ± 0.22	7.66 ± 0.16	**0.012**

^*^Data expressed as mean ± standard deviation

^#^Data expressed as median (*p*_25_–*p*_75_)

A *p* value < 0.05 indicated statistical significance and was highlighted in boldface

**Fig. 5:**
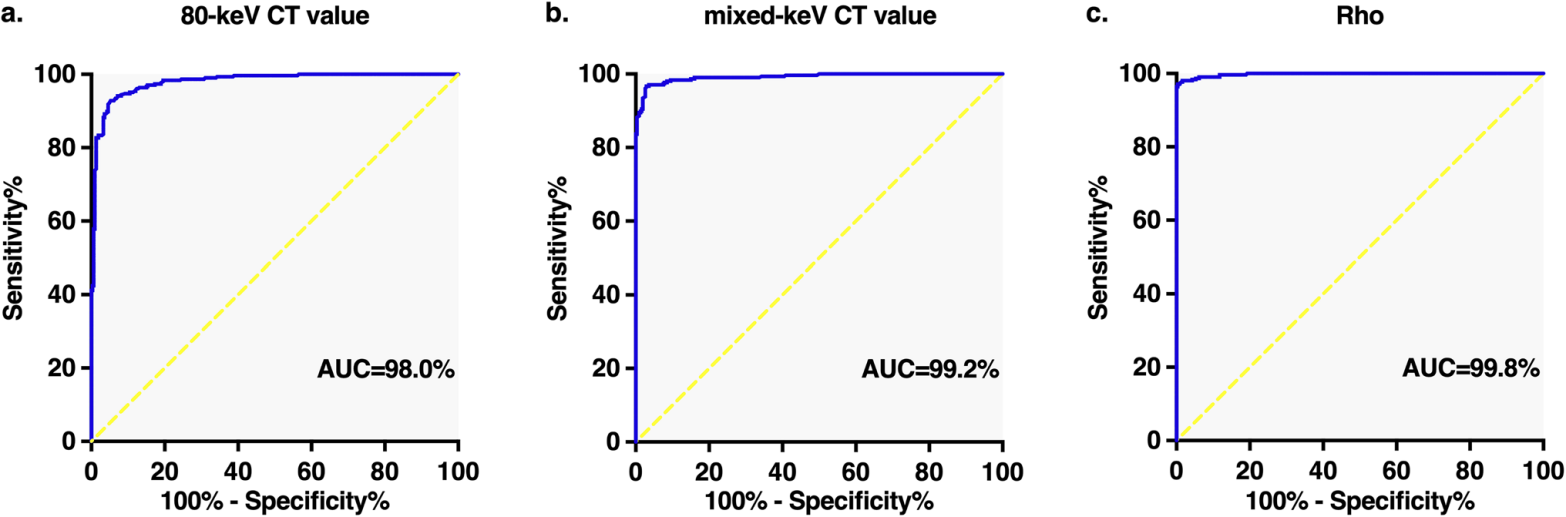
ROC curves of the 80-keV CT value (**a**), mixed-keV CT value (**b**), and Rho (**c**) in the differentiation of normal and torn ACLs

The AUCs for the 80-keV CT value, mixed-keV CT value, and Rho value were excellent, with values of 98.0% (95% CI: 97.0%, 98.9%; cutoff value = 61.8 HU; *p* < 0.001), 99.2% (95% CI: 98.6%, 99.7%; cutoff value = 60.9 HU; *p* < 0.001), and 99.8% (95% CI: 99.6%, 100.0%; cutoff value = 51.8 HU; *p* < 0.001), respectively. In contrast, the Z_eff_ value was not found to be significantly correlated with ACL integrity (*p* = 0.89) (Fig. 5, Tables 3 and 4).

**Table 4:** Validity and reliability of DECT and MRI for the diagnosis of ACL rupture

	DECT			MRI		
	Reader 1	Reader 2	Overall	Reader 1	Reader 2	Overall
Sensitivity (%)^†^	98.0 (50/51) [82.8–100.0]	96.1 (49/51) [79.8–99.9]	97.1 (99/102) [88.1–99.8]	100 (51/51) [86.3–100.0]	98.0 (50/51) [82.8–100.0]	99.0 (101/102) [91.2–100.0]
Specificity (%)^†^	98.0 (50/51) [82.8–100.0]	98.0 (50/51) [82.8–100.0]	98.0 (100/102) [89.5–99.9]	100 (51/51) [86.3–100.0]	100 (51/51) [86.3–100.0]	100 (102/102) [93.0–100.0]
PPV (%)^†^	98.0 (50/51) [89.5–99.9]	98.0 (49/50) [89.4–99.9]	98.0 (99/101) [93.0–99.8]	100 (51/51) [93.0–100.0]	100 (50/50) [92.9–100.0]	100 (101/101) [96.4–100.0]
NPV (%)^†^	98.0 (50/51) [89.5–99.9]	96.2 (50/52) [86.9–99.5]	97.1 (100/103) [91.7–99.4]	100 (51/51) [93.0–100.0]	98.1 (51/52) [89.8–100.0]	99.0 (102/103) [94.7–100.0]
LR^+^	50.0	49.0	49.5	∞	∞	∞
LR^−^	0.02	0.04	0.03	0.00	0.02	0.01
AUC (%)^†^						
80-keV CT value	98.0 [97.0–98.9]			–		
Mixed-keV CT value	99.2 [98.6–99.7]			–		
Rho	99.8 [99.6–100.0]			–		
Z_eff_	n.s					
Accuracy (%)^†^	98.0 (100/102) [93.0–99.7]	97.1 (99/102) [91.7–99.4]	97.5 (199/204) [94.3–99.2]	100 (102/102) [96.4–100.0]	99.0 (101/102) [94.6–100.0]	99.5 (203/204) [97.3–100.0]
*p* value^‡^	> 0.99					

*PPV*, positive predictive value; *NPV*, negative predictive value; *LR*^+^, positive likelihood ratio; *LR*^*−*^, negative likelihood ratio; *AUC*, area under the curve

^†^Data expressed as percentages, with raw data in parentheses and 95% CIs in brackets

^‡^*p* value was obtained using McNemar’s test for the comparison of diagnostic performance between MRI and DECT

Additionally, the measurements exhibited excellent intra- and interobserver reliability (Table 5).

**Table 5:** Intra- and inter-reliability on the measurement of DECT values

	Reader 1		Reader 2	
	ICC	95% CI	ICC	95% CI
Intra-observer (%)				
80-keV CT value	97.9	96.8–98.5	99.0	98.6–99.3
Mixed-keV CT value	99.2	98.9–99.5	99.4	99.2–99.6
Rho	99.0	98.5–99.4	99.0	98.5–99.4
Z_eff_	98.1	97.2–98.7	97.8	96.6–98.5
Inter-observer (%) ^†^				
80-keV CT value	97.5 [96.3–98.3]			
Mixed-keV CT value	96.4 [94.8–97.6]			
Rho	96.9 [95.4–97.9]			
Z_eff_	83.5 [76.5–88.6]			

*ICC*, Intra-class correlation coefficient; *CI*, Confidence interval

^†^Data expressed as percentages, with raw data in parentheses and 95% CIs in brackets

### Diagnostic performance of DECT in detecting ACL ruptures

The validity and reliability of both DECT and MRI in the diagnosis of ACL ruptures were listed in Table 4. The sensitivity was 97.1% (95% CI: 88.1%, 99.8%; 99 of 102 knees) for DECT versus 99.0% (95% CI: 91.2%, 100%; 101 of 102 knees) for MRI; the specificity was 98.0% (95% CI: 89.5%, 99.9%; 100 of 102 knees) for DECT versus 100% (95% CI: 93.0%, 100.0%; 102 of 102 knees) for MRI; and the accuracy was 97.5% (95% CI: 94.3%, 99.2%; 199 of 204 knees) for DECT versus 99.5% (95% CI: 97.3%, 100%; 203 of 204 knees) for MRI. The DECT images had a PPV of 98.0 (95% CI: 93.0%, 99.8%; 99 of 101 knees); an NPV of 97.1% (95% CI: 91.7%, 99.4%; 100 of 103 knees); a mean LR^+^ of 49.5; and a mean LR^−^ of 0.03. McNemar’s test showed no evidence of a difference between the MRI and DECT images for the detection of ACL rupture (*p* > 0.99), based on the average of all readers and the individual readers.

## Discussion

In the present study, we used DECT to evaluate the integrity of ACLs, both healthy and ruptured. The results of this study demonstrated that DECT has excellent validity and reliability for the qualitative and quantitative diagnosis of ACL ruptures, with a reduced 80-keV CT value, mixed-keV CT value, and Rho for torn ACLs (*p* < 0.001).

MRI is the preferred imaging modality with which to visualize and characterize ACLs, regardless of whether they are intact, torn, or reconstructed [11, 13, 22]. However, MRI has disadvantages in acute trauma settings, as well as contraindications for specific participants. Thus, an easily available and reliable substitute imaging examination is needed. In theory, CT has the potential to improve the differentiation of ACLs from adjacent tissues using multiple postprocessing methods; however, CT scans currently provide poor visualization of collagenous structures because of insufficient attenuation contrast and increased beam-hardening artifacts [14, 23]. Conventional CT, therefore, is still limited to the diagnosis of osseous injuries, with rare clinical applications in the assessment of soft tissues. Intriguingly, however, DECT significantly improves the characterization of ligamentous structures, particularly with color-coded postprocessing algorithms and measurable values. Additionally, DECT has the advantages of widespread availability, fast acquisition time, and lower susceptibility to participant motion compared to MRI; therefore, DECT could potentially expedite the diagnosis of ACL rupture, particularly in cases in which there was a high probability that an ACL injury occurred based on initial clinical evaluations.

DECT images were found to have comparable diagnostic validity and reliability to MRI for the diagnosis of ruptured ACLs, based on both qualitative and quantitative methods. Previous studies, as early as 2008, indicated the emerging role of DECT in the visualization of ACLs. Sun et al [24] demonstrated that DECT images could clearly visualize ACLs using multiplanar reformation (MPR) and a volume rendering technique (VRT). In addition to normal ACLs, DECT also qualitatively detected ACL injuries. In a case–control study, 16 torn and 38 intact ACLs were scanned using DECT, after which they underwent postprocessing to generate grayscale, bone removal, and tendon-specific color mapping images [21]. Oblique sagittal images using the bone removal algorithm exhibited almost perfect performance, with a mean AUC > 0.90 for the visualization of injured ACLs in subacute and chronic trauma settings. Similarly, it was found that DECT was a reliable tool for detecting ACL rupture, achieving good sensitivity and specificity in the context of acute trauma [25]. In this study, however, Peltola et al [25] utilized gemstone spectral imaging (GSI) and determined that monochromatic GSI images provided better visualization of cruciate ligaments than bone removal and collagen-specific color mapping images.

In general, tendon-specific color-coded DECT images were not preferred in previous studies. The quality of monochromatic DECT is inferior to that of MRI due to the lower signal-to-noise ratio (SNR) and contrast-to-noise ratio (CNR). Therefore, we innovatively adopted the bone marrow-specific color mapping, in which intact ACLs were highlighted in black and dark red, while torn ACLs were almost or completely uncolored. With the application of this color mapping algorithm, the reliability, accuracy, and convenience were significantly improved when using DECT to qualitatively diagnose ACL injuries. Although Johnson et al [15] initially found that densely packed hydroxylysine and hydroxyproline promoted the differentiation of the main composition of ACLs—collagens—on DECT, there is a dearth of reliable evidence to clarify latent mechanisms of tissue-specific color mapping on ACLs. Moreover, DECT could provide more consecutive images of ACLs, using ≤ 1.0-mm slice thickness and flexible postprocessing methods to better visualize ACLs. It is notable that current studies are limited to quantitative analyses of the diagnostic performance of DECT for evaluating ruptured ACLs. However, we measured the 80-keV CT value, mixed-keV CT value, Rho, and Z_eff_ to quantitatively evaluate the integrity of the ACLs. In the present study, the 80-keV CT value, mixed-keV CT value, and Rho had excellent validity for detecting torn ACLs compared to normal ACLs, with mean AUCs of 98.0%, 99.2%, and 99.8%, respectively. Interestingly, there was no evidence of a difference in the detection of true cases of ACL rupture between DECT and MRI (*p* > 0.99). Therefore, DECT could function as both a qualitative and quantitative diagnostic imaging tool for ACL rupture or as a reliable substitute for MRI in certain conditions, particularly with the application of tissue-specific color mapping.

The present study has two primary strengths. First, the utilization of a bone marrow-specific algorithm improved the differentiation between torn and normal ACLs from a visualized perspective. Second, the bilateral measurements of mixed-keV CT value, 80-keV CT value, and Rho provided a novel quantitative strategy to detect ACL rupture. This study also has some limitations. First, the number of participants enrolled was relatively small. However, based on the results of previous studies, the sample size was calculated and deemed sufficient. We performed a post-hoc power analysis, and > 90% power was attained based on our results. Additionally, the partial volume effect (PVE) may impair the measurement accuracy of these values. Therefore, all knees were scanned using a 1.0 mm slice thickness and were subsequently reconstructed using a 0.75 mm slice thickness to ameliorate the PVE. Moreover, partial ACL injuries were not included in the present study, as all participants were verified to have unrepairable or complete ACL ruptures through intraoperative arthroscopic examination. Therefore, the capability of DECT to distinguish between partial and complete ACL tears requires further investigation. Future studies should also shed light on whether DECT has latent value in evaluating postoperative autograft maturity and radiographic measurements following primary or revision ACL reconstruction.

In conclusion, DECT has the capability to qualitatively and quantitatively diagnose ACL rupture with excellent accuracy and reliability. With the utilization of specific color-mapping algorithms for qualitative assessment, DECT facilitated the visualization of ACLs in an effective and convenient way. Additionally, the 80-keV CT value, mixed-keV CT value, and Rho played quantitative roles in the detection of torn ACLs, with almost perfect AUCs. Furthermore, DECT may become an important imaging modality for evaluating the clinical outcomes ACL reconstruction.

## Abbreviations

ACL: Anterior cruciate ligament
DECT: Dual-energy CT
Rho: Electron density
Z_eff_: Effective atomic number

## Declarations

### Guarantor

The scientific guarantor of this publication is Wenfeng Xiao.

### Statistics and biometry

No complex statistical methods were necessary for this paper.

### Methodology

• prospective

• diagnostic or prognostic study

• performed at one institution
